# Ipilimumab and Stereotactic Radiosurgery with CyberKnife^®^ System in Melanoma Brain Metastases: A Retrospective Monoinstitutional Experience

**DOI:** 10.3390/cancers13081857

**Published:** 2021-04-13

**Authors:** Valentina Borzillo, Rossella Di Franco, Diana Giannarelli, Fabrizio Cammarota, Esmeralda Scipilliti, Emma D’Ippolito, Angela Petito, Marcello Serra, Sara Falivene, Antonio M. Grimaldi, Ester Simeone, Lucia Festino, Vito Vanella, Claudia Trojaniello, Maria Grazia Vitale, Gabriele Madonna, Paolo A. Ascierto, Paolo Muto

**Affiliations:** 1 Radiation Oncology Unit, Istituto Nazionale Tumori-IRCCS-Fondazione G. Pascale, 80131 Naples, Italy; r.difranco@istitutotumori.na.it (R.D.F.); f.cammarota@istitutotumori.na.it (F.C.); esmeralda.scipilliti@istitutotumori.na.it (E.S.); emma.dippolito@istitutotumori.na.it (E.D.); angela.petito@istitutotumori.na.it (A.P.); marcello.serra@istitutotumori.na.it (M.S.); p.muto@istitutotumori.na.it (P.M.); 2 Statistical Unit, Regina Elena National Cancer Institute-IRCCS, 00144 Rome, Italy; diana.giannarelli@ifo.gov.it; 3 Radiation Oncology Unit, Ospedale del Mare, Asl Napoli 1 Centro, 80147 Naples, Italy; sara.falivene@aslnapoli1centro.it; 4 Melanoma, Cancer Immunotherapy and Development Therapeutics, Istituto Nazionale Tumori-IRCCS-Fondazione G. Pascale, 80131 Naples, Italy; a.grimaldi@istitutotumori.na.it (A.M.G.); e.simeone@istitutotumori.na.it (E.S.); l.festino@istitutotumori.na.it (L.F.); v.vanella@istitutotumori.na.it (V.V.); claudia.trojaniello@istitutotumori.na.it (C.T.); mariagrazia.vitale@istitutotumori.na.it (M.G.V.); g.madonna@istitutotumori.na.it (G.M.); p.ascierto@istitutotumori.na.it (P.A.A.)

**Keywords:** stereotactic radiotherapy, radiosurgery, ipilimumab, melanoma brain metastases, CyberKnife

## Abstract

**Simple Summary:**

Retrospective studies have shown a survival advantage in combining ipilimumab with radiotherapy in patients with melanoma brain metastases (MBMs). However, these studies did not clarify the correct timing between the two methods. The aims of our study were to demonstrate the efficacy and toxicity of stereotactic radiotherapy/radiosurgery on MBMs in combination with ipilimumab and estimate the correct timing of treatments to improve patients’ outcomes.

**Abstract:**

The median overall survival (OS) and local control (LC) of patients with melanoma brain metastases (MBMs) are poor even with immune checkpoint inhibitors and/or radiotherapy (RT). The aims of the study were to evaluate the association and timing of stereotactic radiotherapy (SRT)/radiosurgery (SRS) performed with the CyberKnife^®^ System and ipilimumab (IPI). A total of 63 MBMs patients were analyzed: 53 received RT+IPI and 10 RT alone. Therefore, the patients were divided into four groups: RT PRE-PI (>4 weeks before IPI) (18), RT CONC-IPI (4 weeks before/between first and last cycle/within 3 months of last cycle of IPI) (20), RT POST-IPI (>3 months after IPI) (15), and NO-IPI (10). A total of 127 lesions were treated: 75 with SRS (one fraction) and 24 with SRT (three to five fractions). The median follow-up was 10.6 months. The median OS was 10.6 months for all patients, 10.7 months for RT+IPI, and 3.3 months for NO-IPI (*p* = 0.96). One-year LC was 50% for all patients, 56% for RT+IPI, and 18% for NO-IPI (*p* = 0.08). The 1-year intracranial control was 45% for all patients, 44% for RT+IPI, and 51% for NO-IPI (*p* = 0.73). IPI with SRS/SRT in MBMs treatment could improve LC. However, the impact and timing of the two modalities on patients’ outcomes are still unclear.

## 1. Introduction

The brain is frequently a site of metastases in melanoma malignancy, which represents the third most frequent cause of brain metastases after lung and breast cancers [1]. Management of melanoma brain metastases (MBMs) requires a multidisciplinary approach, including local therapies (radiotherapy and surgery) and systemic therapies.

Historically, the median overall survival (OS) of patients with MBMs has been estimated to be 2–5 months [1]. This is probably due to the difficulty of chemotherapy in penetrating the blood–brain barrier (BBB) and the non-enrollment in clinical trials of patients with active MBMs.

Currently as it stands, immune checkpoint inhibitors and molecular target agents, introduced in the treatment of metastatic melanoma, have also been proven effective against brain metastases, leading to median overall survival times of 14 to 23 months [2,3,4,5,6,7].

Local treatments, such as radiotherapy (RT) and surgery, remain important in the management of MBMs and, in combination with new systemic therapies, can improve the outcomes of MBMs patients.

Radiotherapy options comprise stereotactic radiotherapy (SRT)/radiosurgery (SRS) and whole-brain radiotherapy (WBRT), either as single modality treatments or in combination. WBRT is generally delivered to patients with multiple or large-volume inoperable symptomatic lesions, in both the upfront or recurrent setting, even though there is no prospective randomized trial that demonstrates an OS benefit for the addition of WBRT to local therapy. WBRT does not increase survival, with a median OS of 2–5 months and a 1-year survival of less than 13% [8].

In patients with one to four brain lesions and stable extracranial disease, WBRT in combination with SRS or surgery may increase intracranial disease control but can lead to deterioration in neurocognitive functions, damaging neural progenitor cells in the subgranular zone of the hippocampus, with a negative effect on the quality of life of brain metastasis patients [9,10,11]. For this reason, SRS/SRT, with its ability to deliver high doses to the target with simultaneous sparing of the surrounding organs at risk (e.g., brain parenchyma), is used alone without WBRT in this setting of patients [9,10,11,12]. The impact of SRS/SRT on survival and quality of life depends on total dose, fractionation, and multiple variables (size and number of lesions, performance status, extracranial disease status, LDH level) that identify different types of metastatic disease with specific outcomes and prognosis [13,14].

Ipilimumab (IPI), a human monoclonal antibody that blocks the cytotoxic T lymphocyte antigen-4 (CTLA-4) immune checkpoint, was the first agent to show a significant improvement in the median overall survival of patients with extracranial metastatic melanoma in phase III clinical trials [15,16]. The similar intracranial and extracranial response rates obtained from ipilimumab in prospective clinical trials suggest that ipilimumab may not be limited by the BBB disrupted by MBMs [6,15,17,18,19,20]. Ipilimumab has activity in some patients with MBMs particularly when metastases are small and asymptomatic [17]. Queirolo et al. reported the outcome of 146 patients with asymptomatic brain metastases who received treatment with second-line ipilimumab, and the median OS was 4.3 months and the 1-year OS was 20% [20]. The outcome rates with ipilimumab alone or in combination with fotemustine in MBMs patients appear to be significantly lower in comparison with SRS alone (median OS of 8–11 months, 1-year LC rates of 49%–75%) or local-treatment (SRS/SRT/WBRT/surgery)-combined modality (median OS of 13–14 months) [3,6,13,19,20,21,22,23].

Retrospective analysis demonstrates an advantage in median OS with the association of ipilimumab and radiotherapy in patients with MBMs, without however clarifying the optimal timing of these two modalities [24,25,26,27,28,29,30,31,32,33,34,35,36]. In this monoinstitutional retrospective analysis, we reported efficacy and toxicity data from patients with MBMs treated with the SRS/SRT CyberKnife^®^ (CK) System with or without ipilimumab.

## 2. Materials and Methods

### 2.1. Patients and Methods

Sixty-three consecutive patients with MBMs were treated with CyberKnife^®^ SRS/SRT from December 2012 to December 2018. The patients were grouped into those who had received radiotherapy and ipilimumab (RT+IPI) and those who had received radiotherapy alone (NO-IPI). The RT+IPI group was also divided into three different subgroups based on the timing of the therapies: (1) patients who received radiotherapy more than 4 weeks before day 1 of the first cycle of IPI (RT PRE-IPI), (2) patients who received radiotherapy 4 weeks before day 1 of the first cycle of IPI or between day 1 of the first cycle and day 1 of the last cycle of IPI or within 3 months of day 1 of the last cycle (RT CONCOMITANT (CONC)-IPI), and (3) patients who received radiotherapy more than 3 months after day 1 of the last cycle of IPI (RT POST-IPI). Ipilimumab was administered intravenously at a dose of 3 mg/kg every 3 weeks for four doses. A corticosteroid therapy was administered to all patients during RT (dexamethasone, 8 mg/daily for 3–5 days), and then at decreasing doses over the following weeks. The patients were evaluated with clinical and instrumental follow-up with brain contrast-enhanced magnetic resonance imaging (MRI) with a thin section (1 mm) at 2 months after radiotherapy and every 2 months for the first year and every 3 months thereafter. In case of appearance of new intracranial lesions or progression of a treated lesion, the treatment options were SRS, WBRT, or surgery according to the number of lesions, the patient’s performance status, and the extent of extracranial disease. Radionecrosis and intratumoral hemorrhage were evaluated by brain perfusion-weighted imaging (PWI) MRI performed during follow-up. OS was considered from the date of the first SRS/SRT treatment to the date of death (event) or last follow-up (censored). Local response was evaluated on brain MRI performed during follow-up using response evaluation criteria in solid tumors (RECIST) and revised using response criteria for brain metastases from the response assessment in neuro-oncology criteria (RANO) group [37,38]. Local control (LC) (in the SRS/SRT field) was defined as the sum of complete response (CR), partial response (PR), and stable disease (SD) of a single lesion treated from the date of the SRS/SRT to the date of the last follow-up. Intracranial control (IC) (out of the SRS/SRT field) was defined as the absence of new lesions outside the SRS field on brain MRI performed during follow-up.

### 2.2. Radiotherapy Technique

All patients were treated in supine position. Immobilization was performed using a customized thermoplastic head mask. A simulation computed tomography (CT) scan (1 mm slice thickness) with contrast enhancement was performed for treatment plan calculation. Before SRS/SRT, all patients performed a diagnostic brain contrast-enhanced magnetic resonance imaging (MRI) (T1–T2w) with a thin section (1–1.5 mm). Image fusion with diagnostic MRI sequences was performed and used for the identification and delineation of target volume and organs at risk (OARs). The clinical target volume (CTV) was the same volume of the gross tumor volume (GTV), and the planning target volume (PTV) was the CTV plus an isotropic 1.5–2 mm margin. Contoured OARs were whole brain, left and right (L–R) eye, L–R lens, L–R optic nerve, optic chiasm, pituitary gland, brainstem, L–R cochlea, and spinal cord. Total dose was prescribed to the 80% isodose line. Total dose and fractionation were based on size (<2 cm, 2–3 cm, >3 cm), location of the lesion, previous RT treatment, and the patient’s performance status. SRS was defined as a single fraction treatment, and SRT as a greater-than-one-but-not-more-than-five fraction treatment. We treated with SRS/SRT only patients with ≤4 brain metastases. Multiplan Software (Accuracy Inc., Sunnyvale, CA, USA) was used for the treatment plan. Radiotherapy was performed by the CyberKnife^®^ System, a frameless image-guided robotic radiosurgery system [27] composed of a small linear accelerator (6 MV photon) mounted on a robotic arm, which moves around the patient, changing the position according to the movement of the patient with millimetric accuracy.

### 2.3. Statistical Analysis

Time-to-event curves were calculated with the Kaplan–Meier method and compared with the log-rank test. The Cox proportional hazards model was used to assess hazard ratios (HR) with 95% confidence intervals (95% CI) and to assess the independent prognostic values of the variables considered. Multivariate analysis was performed using a stepwise selection procedure. The results presented using IBM SPSS, version n.20, (IBM, Rome, Italy) were used for the analysis.

## 3. Results

Patient, tumor, and treatment characteristics are summarized in Table 1.

A total of 63 patients were divided into four groups: 18 in the RT PRE-IPI group, 20 in the RT CONC-IPI group, 15 in the RT POST-IPI group, and 10 in the NO-IPI group. Six of the 53 patients did not perform all four ipilimumab cycles: 5 patients died from progression of disease (after one cycle (*n* = 3), two cycles (*n* = 1), and three cycles (*n* = 1)), and 1 patient discontinued for toxicity after two cycles. All systemic therapies performed before and after SRS/SRT are summarized in Table 2.

**Figure 1:**
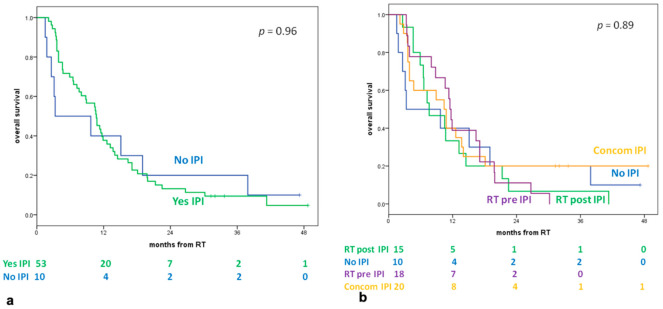
(**a**) Overall survival according to ipilimumab (IPI). (**b**) Overall survival according to four groups. Legend: RT: Radiotherapy; Yes IPI: patients who received radiotherapy and Ipilimumab; NO IPI: patients which received only Radiotherapy; RT POST-IPI: patients who received radiotherapy more than 3 months after day 1 of the last cycle of IPI; RT PRE-IPI: patients who received radiotherapy more than 4 weeks before day 1 of the first cycle of IPI; CONCOM-IPI: patients who received radiotherapy 4 weeks before day 1 of the first cycle of IPI or between day 1 of the first cycle and day 1 of the last cycle of IPI or within 3 months of day 1 of the last cycle.

The median OS for all patients was 10.6 months (95% CI; 8.5–12.7), and the median OS of 53 patients who received IPI and RT was 10.7 months (95% CI; 8.2–13.1) vs. 3.3 months (95% CI; 0–13.5) for the 10 patients in the NO-IPI group (*p* = 0.96) (Figure 1a). The median OS for a single group was 7.6 months (95% CI; 2.7–12.5) for RT POST-IPI, 10.4 months (95% CI; 7.6–13.2) for RT CONC-IPI, and 11.5 months (95% CI; 10.7–12.3) for RT PRE-IPI (*p* = 0.89) (Figure 1b).

The 1- and 2-year OSs of all patients were 38% and 14%, respectively. The 1- and 2-year OSs were 40% and 20%, 33% and 7%, 40% and 20%, 39% and 11% for the NO-IPI, RT POST-IPI, RT CONC-IPI, and RT PRE-IPI groups, respectively.

On univariate analysis, OS was associated with Eastern Cooperative Oncology Group Performance Status (ECOG PS) (*p* = 0.005), time from diagnosis to brain metastases (*p* = 0.02), LDH values (*p* = 0.005), and lesion size (*p* = 0.004). At the multivariate analysis, LDH values (*p* = 0.003) and lesion size (*p* = 0.001) were both significantly associated with OS (Table 3).

At the time of analysis, 5 out of 63 patients were still alive: 4 in the RT CONC-IPI group and 1 in the NO-IPI group. The median follow-up was 10.6 months (range, 1.5–48.7 months). Fifty-nine patients and 123 lesions were evaluable for the follow-up.

**Figure 2:**
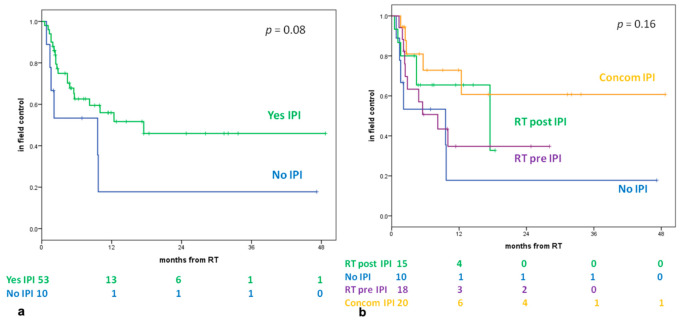
(**a**) Local control (in the SRS field) according to IPI. (**b**) Local control (in the SRS field) according to four groups. Legend: RT: Radiotherapy; Yes IPI: patients who received radiotherapy and Ipilimumab; NO IPI: patients which received only Radiotherapy; RT POST-IPI: patients who received radiotherapy more than 3 months after day 1 of the last cycle of IPI; RT PRE-IPI: patients who received radiotherapy more than 4 weeks before day 1 of the first cycle of IPI; CONCOM-IPI: patients who received radiotherapy 4 weeks before day 1 of the first cycle of IPI or between day 1 of the first cycle and day 1 of the last cycle of IPI or within 3 months of day 1 of the last cycle.

The 1-year LC (in the SRS/SRT field) of all patients’ lesions treated was 50%, and the 1-year LC of patients who received IPI and RT was 56% vs. 18% for patients who did not receive IPI (*p* = 0.08) (Figure 2a). The 1- and 2-year LCs (in the SRS/SRT field) for a single group were 18% for the NO-IPI group, 65% and 33% for the RT POST-IPI group, 73% and 61% for the RT CONC-IPI group, and 35% for the RT PRE-IPI group, respectively (*p* = 0.16) (Figure 2b).

In a multivariate Cox model, the size of the lesion (<8 mm) (*p* = 0.01) and the timing of IPI–RT (*p* = 0.005) were both independently associated with better LC (Table 4).

The 1-year intracranial control (out of the SRS/SRT field) of all patients was 45%, and the 1-year IC of patients who received IPI and RT was 44% vs. 51% for patients who did not receive IPI (*p* = 0.73). The 1-year IC for a single group was 51% for the NO-IPI group, 47% for the RT POST-IPI group, 54% for the RT CONC-IPI group, and 31% for the RT PRE-IPI group (*p* = 0.44).

**Figure 3:**
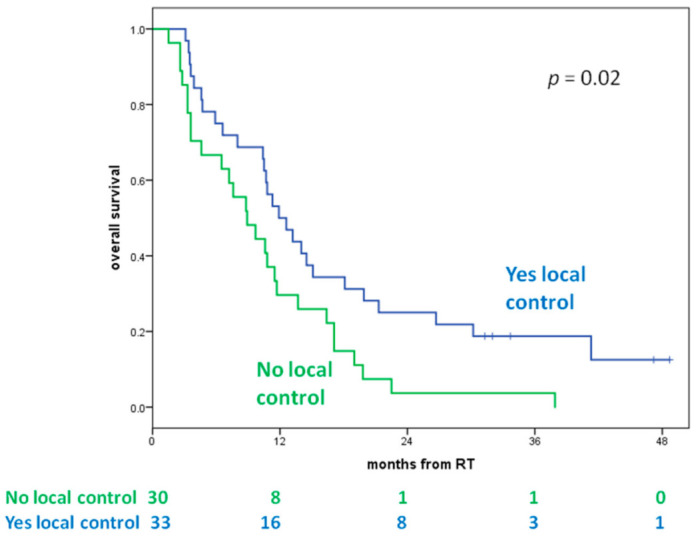
Overall survival according to local control. No local control: patients who did not achieve local control; Yes local control: patients who achieved local control.

The 1- and 2-year OSs of patients with LC were 50% and 25% vs. 30% and 4% for patients without LC, respectively (*p* = 0.02) (Figure 3).

After CyberKnife SRS/SRT, 15 patients with intracranial progression received WBRT (30 Gy in 10 fractions); 2 patients from the NO-IPI group were retreated with SRS on the same lesion treated 10 months previously; 23 patients received SRS/SRT treatment for new lesions (one to three lesions per patient); and 2 patients underwent surgery, 1 after 4 and the other 1 after 14 months from SRS/SRT. The median interval between the first and subsequent SRS treatments was 8 months (range, 2–35 months).

Intratumoral hemorrhage was observed in 10 patients: in 1 patient (NO-IPI group) 30 days after SRS/SRT, in 7 patients (2 RT CONC-IPI, 2 RT POST-IPI, 3 RT PRE-IPI groups) after 60 days from RT, in 1 patient (RT PRE-IPI group) after 90 days from RT, and in 1 patient (NO-IPI group) after 10 months. Radionecrosis was observed in 4 patients.

## 4. Discussion

For many years, the management of MBMs comprised surgery, radiotherapy, and a small number of chemotherapeutic agents. Chemotherapy alone has been proven to be ineffective for the treatment of MBMs with a median survival of 2.2 months due to the inability to penetrate across the BBB [40,41]. Not even temozolomide, which crosses the BBB, in combination with WBRT, has proven its effectiveness in MBMs patients [42]. Therefore, due to lack of effective therapies and poor prognosis, patients with MBMs have long been excluded from clinical trials. However, with the advent of new agents (immune checkpoint inhibitors (ICIs) and BRAF inhibitors) and studies confirming that T cells can cross the BBB, the situation has changed [43,44].

Hodi et al. [15], analyzing metastatic melanoma patients receiving ipilimumab, supported two hypotheses: ipilimumab, with the BBB disrupted by MBMs, may cross into the perivascular space and activate peripherally recruited T cells or IPI-activated T cells in the peripheral circulation entering at porous sites and targeting MBMs. This and other studies, which achieved similar intra- and extracranial response rates, suggest that IPI may not be limited by the BBB and has similar benefits and toxicity profile in patients with and without BMs [15,18,19,20,24].

Radiotherapy, indeed, in studies with mouse melanoma models, seems to optimize the systemic antitumor immune response induced by immunotherapy and prevent cancer cells from evading immune response via several mechanisms (e.g., activated and increased T cell infiltration after radiotherapy-induced cell death) [45,46,47,48]. The synergistic effect of RT plus immunotherapy may explain the regression of tumors outside the radiation target field, the so-called “abscopal effect”, found both in murine models and in patients treated with IPI and RT [30,49,50,51].

Several retrospective studies have shown how the combination of high-dose RT (SRS/SRT) and ipilimumab in the treatment of MBMs patients improves outcomes, without however clarifying which is the best timing for the association of the two treatments [25,26,27,28,29,36].

Two retrospective studies showed a clear advantage in terms of median OS with the use of SRS+IPI compared with RT alone (21.3 vs. 4.9 months and 19.9 vs. 4 months) in the treatment of MBMs. However, both studies failed to obtain a statistical significance in terms of median survival in the RT PRE-IPI vs. RT POST-IPI group analysis (21.3 vs. 19.8 months and 18.4 vs. 8.1 months) and therefore to give an answer about the right timing [25,26].

Schoenfeld [35], in a review of 16 cases of melanoma with brain metastases treated with RT+IPI, demonstrated an advantage in terms of OS in favor of radiotherapy performed before receiving ipilimumab (26 months) compared with RT post-IPI (6 months) or IPI and RT concurrently (18 months).

However, these findings should be interpreted with caution considering that this study was performed in a very small cohort of 16 patients and with two different RT techniques (SRS and WBRT). Some retrospective studies have described in detail the criteria for dividing patients into subgroups (CONCOMITANT, PRE, or POST) based on the timing (days or months) of IPI and RT administration; these criteria are different in each of these studies. Kiess et al. [27] divided patients into three groups: “SRS before IPI”, who received SRS before the first dose of IPI; “SRS during IPI”, who received SRS between doses of IPI or <1 month after the last dose of IPI; and “SRS after IPI”, who received SRS >1 month after the last dose of IPI. The study demonstrated a similar benefit in OS in SRS during (1-year OS, 65%) and before IPI (1-year OS, 56%) groups versus SRS after IPI (1-year OS, 40%) with favorable median OS for all patients of 12.4 months (range, 2–89 months). Patel et al. [29] specified the timing of IPI and SRS of 20 MBMs patients treated: 7 (35%) patients received IPI within 14 days of SRS, whereas 13 (65%) received IPI >14 days of SRS, but within 4 months. The higher 1- and 2-year OS rates (42.9% and 42.9%, respectively) in IPI within 14 days with respect to IPI >14 days of SRS (33.8% and 16.9%) and SRS alone (38.5% and 25.7%) were demonstrated but without statistical significance.

Skrepnik et al. [32] evaluated 25 patients with 58 MBMs and found better brain control in patients who received SRS during and within 30 days (before or after) of ipilimumab infusion, also resulting in better survival. Cohen-Inbar et al. [33] analyzed 46 MBMs patients divided into two groups based on the timing of SRS and IPI treatment: 32 patients (28 treated with SRS before the first dose of IPI and 4 with SRS during IPI or within 1 month of the last IPI dose) constituted group A, and another 14 patients (treated with SRS more than 1 month after completing IPI treatment) constituted group B. The results showed an improvement in the LC of a previously treated area in group A (median, 19.6 months) compared with group B (median, 3 months) (*p* = 0.002), with more post-SRS perilesional edema in group A.

A retrospective multi-institutional analysis was conducted on 99 patients (71 MD Anderson and 28 Yale–New Haven Hospital) with MBMs treated with SRS after last dose of IPI (within or after 5.5 months) [34]. The study concluded that, in the MD Anderson cohort, patients who received SRS within 5.5 months of IPI therapy (*n* = 51) had better intracranial control than those (*n* = 20) who received SRS later (median, 8.43 vs. 3.63 months, *p* = 0.02), with no statistically significant difference in median OS between the two arms. The improvement in intracranial control was confirmed by an independent validation Yale cohort. All retrospective studies listed above have shown that RT performed 14 days, 30 days, within 1 month, or 5.5 months before or after IPI can improve the OS or LC of a previously irradiated area. However, these experiences have the limitation of being retrospective, with few and heterogeneous patients, with different timings of the combination IPI–RT and with different types of radiation techniques (WBRT or SRS).

Our study seems to show a slight advantage in median OS by the use of the combination RT+IPI (10.7 months) compared with RT alone (3.3 months) even if the results are not statistically significant. Furthermore, the study did not obtain statistically significant survival data for a single group (11.5 months in RT PRE-IPI, 10.4 months in RT CONC-IPI, and 7.6 months in RT POST-IP). However, survival rates seem to demonstrate an advantage in the IPI CONC group over other groups mostly at 2 years (40% and 20% vs. 33% and 7% of the RT POST-IPI group vs. 39% and 11% of the RT PRE-IPI group, respectively, at 1–2 years). Univariate analysis showed that a good performance status (ECOG 0), a time between primary and brain metastasis diagnosis greater than 30 months, normal LDH values, and lesion size <8 mm are associated with better survival. Normal LDH values and lesion size are also significant in the multivariate analysis.

Our results seem to show a slight advantage in 1-year LC (in the SRS/SRT field) of patients treated with ipilimumab and SRS/SRT compared with those treated with RT alone (56% vs. 18%, respectively) even if the results are not statistically significant (*p* = 0.08). These data could be the result of possible synergism between immunotherapy and radiotherapy on the site of irradiation. The analysis for single groups shows a slight advantage on LC (in the SRS/SRT field) at 1 to 2 years in the RT CONC group (73% and 61% vs. 65% and 33% for the RT POST-IPI group and 35% for the RT PRE-IPI group, respectively), even if the results are not statistically different (*p* = 0.16). In a multivariate analysis, the size of lesion <8 mm and the timing of IPI–RT were both independently associated with better LC. No statistically significant differences were found in terms of 1-year intracranial control in the analysis of RT+IPI and NO-IPI groups (44% vs. 51%) and with the single-group analysis (47% RT POST-IPI group, 54% RT CONC-IPI group, and 31% RT PRE-IPI group (*p* = 0.44). Intracranial control probably depends primarily on the behavior of a systemic disease and its response to therapies. The combination of IPI and SRS can also have an impact on local and intracranial controls, although there are conflicting data in the literature in relation to correct timing [26,27,29,36].

## 5. Validity and Limitation of the Study

In our experience, 63 MBMs patients received the same RT technique SRS/SRT with the CyberKnife^®^ System.

A total of 58 of 63 patients received SRS/SRT as upfront treatment without adjuvant WBRT in order to avoid neurocognitive morbidity associated with WBRT and to administer high doses per fraction. This probably led to a lower control out of the SRS field and in 23 patients, making it necessary to perform additional radiosurgery treatment for new lesions at the median interval between the first and subsequent SRS of 8 months (range, 2–35 months). It is interesting to note in our study that patients who achieved good LC had 1- and mostly 2-year OS rates greater than patients without LC (50% and 25% vs. 30% and 4%, respectively; *p* = 0.02).

In our experience, we found a low rate of radionecrosis (4 patients). This could be related to the high accuracy of the CyberKnife^®^ System, which allows for sparing healthy brain parenchyma, or at the patient’s death before the occurrence of the event.

The small number of patients (63) limits the statistical power of the analysis among the subgroups.

Other limitations are the retrospective nature of the study and the absence of an IPI-only group as control. It is not clear whether it was the LC obtained with RT+IPI that contributed to increased survival or the different systemic therapies performed on the patients (e.g., BRAF inhibitors and/or ICI).

There are no clear data in our study on the impact of the combination IPI and RT on survival, because of selection bias. However, our results seem to show that RT associated with IPI can improve LC in the SRS/SRT field. In addition, our data show that patients who obtain greater LC also have greater survival and improved quality of life.

The results show a trend in favor of the RT CONC-IPI group in terms of 2-year OS (Figure 1b).

## 6. Conclusions

The combination of IPI and SRS/SRT in MBMs patients can increase the LC of brain lesions treated, and patients who obtain greater LC have better survival. However, the optimal timing of the combination of these two approaches is still unclear. Instead seems to be an advantage in terms of LC when radiotherapy is performed concomitantly with IPI. The combination RT–IPI was found to be feasible with a low toxicity profile and a low radionecrosis rate. Further prospective studies are needed to determine the optimal sequence of RT and checkpoint inhibitor treatment and its real impact on outcomes in MBMs patients.

## Figures and Tables

**Table 1:** Patient and treatment characteristics.

		NO-IPI (10 pts.)	RT POST-IPI (15 pts.)	RT CONC-IPI (20 pts.)	RT PRE-IPI (18 pts.)	TOTAL (63 pts.)
Sex	M F	7 3	5 10	9 11	13 5	34 29
Age (years)	Median Range	64 40–77	62 29–81	55 28–80	63 32–80	60 28–81
ECOG PS	0 1	8 2	14 1	17 3	14 4	53 10
RPA	Class I Class II	0 10	0 15	1 19	3 15	4 59
DS-GPA [39]	1 2 3 4	0 1 4 5	0 3 6 6	0 3 7 10	0 0 10 8	0 7 27 29
Melanoma site	Cutaneous Mucosal Unknown Ocular	8 1 1 0	14 1 0 0	18 1 0 1	17 0 1 0	57 3 2 1
Time between diagnosis and BMs (months)	Median Range	34 0–192	37 0–240	23 0–228	34 3–240	34 0–240
Extracranial disease	Yes No	6 5	23 1	6 1	14 7	49 14
LDH pre-RT	Normal High NA	4 4 2	9 5 1	11 7 2	9 6 3	33 22 8
BRAF status	Mutated Wild type NA	8 2	5 10	7 13	9 8 1	29 33 1
Neurological symptoms	Asymptomatic Symptomatic	5 5	15 0	14 6	14 4	48 15
Steroid treatment pre-RT	Yes No NA	5 4 1	5 8 2	7 13 -	9 9 -	26 34 3
Number of BMs treated		16	34	38	39	127
Lesion size	Median (mm) Range (mm) 0–2 (cm) >2–<3 (cm) >3 (cm) NA	9 2–30 13 3 0-	9 3–36 28 3 2 1	8 2–42 33 3 2 -	8 3–37 31 7 1 -	8 2–42 105 16 5 1
Radiation Treatment	SRS (10–24 Gy) SRT (18–24 Gy)	11 4	20 4	21 6	21 10	75 24
Treatments before CK SRS/SRT	SRS WBRT Surgery	0 2 5	1 2 0	0 0 0	0 1 1	1 5 6
Treatments after CK SRS/SRT	SRS/SRT * WBRT Surgery	3 # 0 1	7 4 0	7 4 0	8 7 1	25 15 2

Legend: IPI: ipilimumab; RT: Radiotherapy; NO IPI: patients which received only Radiotherapy; RT POST-IPI: patients who received radiotherapy more than 3 months after day 1 of the last cycle of IPI; RT CONC-IPI: patients who received radiotherapy 4 weeks before day 1 of the first cycle of IPI or between day 1 of the first cycle and day 1 of the last cycle of IPI or within 3 months of day 1 of the last cycle; RT PRE-IPI: patients who received radiotherapy more than 4 weeks before day 1 of the first cycle of IPI; ECOG PS: Eastern Cooperative Oncology Group Performance Status; RPA: recursive partitioning analysis: DS-GPA: diagnosis-specific graded prognostic assessment; BMs: brain metastases; LDH: lactate dehydrogenase; NA: not assessed; CK: CyberKnife^®^; SRS: radiosurgery; SRT: stereotactic radiotherapy; WBRT: whole brain radiotherapy; * Number of treatments after first CK SRS/SRT; # two retreatments on the same lesion treated with first CK SRS/SRT; NA: not available; pts: patients; SRS: stereotactic radiosurgery; SRT: stereotactic radiotherapy; CK: CyberKnife; WBRT: whole-brain radiotherapy.

**Table 2:** Systemic therapies performed before and after SRS/SRT.

	Therapies before SRS/SRT					Therapies after SRS/SRT				
Groups	NO IPI	RT POST	RT CONC	RT PRE	TOT	NO IPI	RT POST	RT CONC	RT PRE	TOT
Any Immunotherapy	1(10) *	15 (100)	2 (10)	3 (17)	21	1 (10)	7 (47)	11 (55)	18 (100)	37
IPI		15 (100)			15		2 (13)		18 (100)	20
Anti-PD1	1 (10)	7 (47)	1 (5)	1 (6)	10	1 (10)	5 (33)	11 (55)	4 (22)	21
Interferon			1 (5)	2 (11)	3					
BRAF inhibitor or MEK inhibitor	3 (30)	2 (13).	1 (5)	5 (28)	11			1 (5)	2 (11)	3
BRAF + MEK inhibitor	2 (20)	2 (13)	2 (10)	3 (17)	9	1 (10)		5 (25)	1 (6)	7
Chemotherapy	3 (30)	11(73)	5 (25)	6 (33)	25		2 (13)	4 (20)	3 (17)	9

Legend: SRS: radiosurgery; SRT: stereotactic radiotherapy; IPI: ipilimumab; RT: Radiotherapy; NO IPI: patients which received only Radiotherapy; RT POST-IPI: patients who received radiotherapy more than 3 months after day 1 of the last cycle of IPI; RT CONC-IPI: patients who received radiotherapy 4 weeks before day 1 of the first cycle of IPI or between day 1 of the first cycle and day 1 of the last cycle of IPI or within 3 months of day 1 of the last cycle; RT PRE-IPI: patients who received radiotherapy more than 4 weeks before day 1 of the first cycle of IPI; TOT: total; PD1: programmed cell death protein 1; * Number in brackets is expressed in percentage.

**Table 3:** Univariate and multivariate analysis for overall survival.

	Univariate		Multivariate	
	Hazard Ratio (95% CI)	*p*-Value	Hazard Ratio (95% CI)	*p*-Value
Gender Male vs. female	1.01 (0.60–1.70)	0.97		
Age ≥61 years vs. <61 years	1.38 (0.82–2.32)	0.23		
ECOG PS PS 1 vs. PS 0	2.71 (1.35–5.46)	0.005	--	
DS-GPA [39] 4 vs. 2–3	0.64 (0.38–1.08)	0.09		
Time to brain mets >30 months vs. <30 months	0.53 (0.31–0.92)	0.02	--	
*N* of brain mets treated 2 vs. 1 >3 vs. 1	0.70 (0.38–1.31) 0.80 (0.43–1.51)	0.51		
Extracranial disease Yes vs. no	1.61 (0.86–2.99)	0.13		
LDH pre-RT (*) Elevated vs. normal	2.34 (1.30–4.23)	0.005	2.54 (1.37–4.73)	0.003
BRAF Mutated vs. WT	1.00 (0.59–1.70)	0.99		
Ipilimumab RT POST vs. NO IPI CONC RT vs. NO IPI RT PRE vs. NO IPI	1.14 (0.49–2.62) 0.86 (0.38–1.95) 1.00 (0.44–2.26)	0.89		
*N* of RT >1 vs. 1	0.82 (0.47–1.43)	0.48		
Lesion size (maximum diameter) (mm) (>8 vs. ≤8 mm)	2.56 (1.36–4.81)	0.004	3.25 (1.58–6.64)	0.001
Radiotherapy (SRS vs. SRT)	0.67 (0.39–1.15)	0.15		

Legend: ECOG PS: Eastern Cooperative Oncology Group Performance Status: DS-GPA: diagnosis-specific graded prognostic assessment; LDH: lactate dehydrogenase; IPI: ipilimumab; RT: Radiotherapy; NO IPI: patients which received only Radiotherapy; RT POST-IPI: patients who received radiotherapy more than 3 months after day 1 of the last cycle of IPI; RT CONC-IPI: patients who received radiotherapy 4 weeks before day 1 of the first cycle of IPI or between day 1 of the first cycle and day 1 of the last cycle of IPI or within 3 months of day 1 of the last cycle; RT PRE-IPI: patients who received radiotherapy more than 4 weeks before day 1 of the first cycle of IPI; SRS: radiosurgery; SRT: stereotactic radiotherapy; * LDH is not known in all patients.

**Table 4:** Univariate and multivariate analysis for local control of the treated lesions.

	Univariate		Multivariate	
	Hazard Ratio (95% CI)	*p*-Value	Hazard Ratio (95% CI)	*p*-Value
Ipilimumab RT POST vs. NO IPI CONC RT vs. NO IPI RT PRE vs. NO IPI	0.26 (0.12–0.54) 0.45 (0.13–1.58) 0.55 (0.27–1.09)	0.005	0.25 (0.12–0.53) 0.42 (0.12–1.48)0.48 (0.24–0.96)	0.005
Lesion size (maximum diameter) (mm) (>8 vs. ≤8 mm)	2.05 (1.16–3.64)	0.01	2.14 (1.21–3.80)	0.01

Legend: IPI: ipilimumab; RT: Radiotherapy; NO IPI: patients which received only Radiotherapy; RT POST-IPI: patients who received radiotherapy more than 3 months after day 1 of the last cycle of IPI; RT CONC-IPI: patients who received radiotherapy 4 weeks before day 1 of the first cycle of IPI or between day 1 of the first cycle and day 1 of the last cycle of IPI or within 3 months of day 1 of the last cycle; RT PRE-IPI: patients who received radiotherapy more than 4 weeks before day 1 of the first cycle of IPI.

## Data Availability

The authors declare the availability of data and materials.
